# Characterization of five-year observation data of fine particulate matter in the metropolitan area of Lahore

**DOI:** 10.1007/s11869-017-0464-1

**Published:** 2017-03-14

**Authors:** Fatima Khanum, Muhammad Nawaz Chaudhry, Prashant Kumar

**Affiliations:** 1 College of Earth and Environmental Sciences, The University of Punjab, Lahore, Pakistan; 2 Department of Civil and Environmental Engineering, Faculty of Engineering and Physical Sciences, University of Surrey, Guildford, GU2 7XH UK; 3 Environmental Flow (EnFlo) Research Centre, Faculty of Engineering and Physical Sciences, University of Surrey, Guildford, GU2 7XH UK

**Keywords:** Fine particles, Air quality monitoring, Meteorological parameters, Criteria pollutants, Health risk

## Abstract

This study aims to assess the long-term trend of fine particles (PM_2.5_; ≤2.5 μm) at two urban sites of Lahore during 2007–2011. These sites represent two distinct areas: commercial (Townhall) and residential cum industrial (Township). The highest daily mean concentrations of PM_2.5_ were noted as 389 and 354 μg m^−3^ at the Townhall and Township sites, respectively. As expected, the annual seasonal mean of PM_2.5_ was about 53 and 101% higher during winter compared with the summer and monsoon/post-monsoon seasons, respectively. On contrary to many observations seen in developing cities, the annual mean PM_2.5_ during the weekends was higher than weekdays at both monitoring sites. For example, these were 100 (142) and 142 μg m^−3^ (148) during the weekdays (weekends) at the Townhall and Township sites, respectively. The regression analysis showed a significant positive correlation of PM_2.5_ with SO_2_, NO_2_ and CO as opposed to a negative correlation with O_3_. The bivariate polar plots suggested a much higher influence of localized sources (e.g., road vehicles) at the Townhall site as opposed to industrial sources affecting the concentrations at the Township site. The imageries from the MODIS Aqua/Terra indicated long-range transport of PM_2.5_ from India to Pakistan during February to October whereas from Pakistan to India during November to January. This study provides important results in the form of multiscale relationship of PM_2.5_ with its sources and precursors, which are important to assess the effectiveness of pollution control mitigation strategies in Lahore and similar cities elsewhere.

**Figure:**
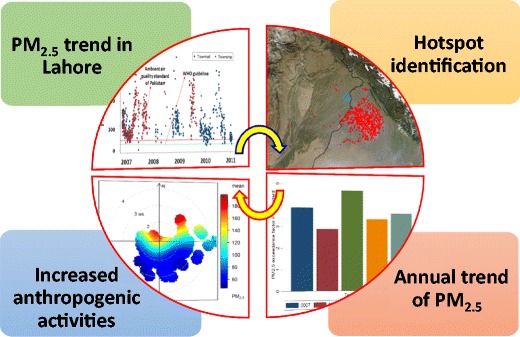
Graphical abstract

Graphical abstract

## Introduction

Lahore is a metropolitan area with high levels of particulate pollution that often surpasses the guideline values of World Health Organization (WHO) and the National Ambient Air Quality Standards (NAAQS) of Pakistan (Pak-EPA 2005). Both fine and coarse particulate matter cause various types of health concerns (e.g., Stone et al. 2010; Kim et al. 2011; Tsiouri et al. 2015; Lan et al. 2016). The WHO estimated ∼360,000 premature deaths in Asia each year due to air pollution (WHO 2008). The environmental degradation, including water and soil, is about 6% of Pakistan’s GDP, and the indoor and outdoor air pollution contributes nearly half of it towards the total illness and premature mortality (World Bank 2006). The lack of stringent implementation of air pollution regulations and the mass transportation system contribute further to the issue of local air pollution (Biswas et al. 2008). Needless to mention that the particulate matter (PM) also plays an important role in affecting the global climate (IPCC 2007; Karagulian et al. 2015).

The increasing population and urbanization have led to an increase in numerous industrial sources as well as the road vehicles (Biswas et al. 2008; Stone et al. 2010; Shah et al. 2012; Rasheed et al. 2015; Ali et al. 2015; Molina et al. 2017). New evidence related to exposure risk assessment and global exposure estimates shows that the exposure to the ambient PM has increased than previously estimated (WHO 2014a). In megacities such as Lahore, important factors for the increased exposure to air pollution are the higher intensity of human activities and emissions from the road vehicles. PM is currently considered to be one of the best indicators for assessing health impacts caused by the ambient air pollution (WHO 2014a; Yao et al. 2015).

Air pollution control in Pakistan has not yet become an electoral issue due to a lack of adequate information for decision and policy makers (Shah et al. 2012), although some sporadic reports recognize airborne PM as a serious environmental and health concern in urban areas of Pakistan (Colbeck et al. 2010; Pak-EPA 2005). As summarized in Table 1, previous studies of ambient air quality in Lahore have documented 24-h averaged maximum PM_2.5_ during winter season as 200 μg m^−3^ (Biswas et al. 2008), springtime PM_10_ as 460 μg m^−3^ (Zhang et al. 2008a) and total suspended PM well above 900 μg m^−3^ (Ghauri et al. 2007).

**Table 1:** Summary of the past selected PM studies carried out in Pakistan

Location	PM types	Concentration (μg m^−3^)	Time span	Reference
Lahore (roadside monitoring)	PM_10_	895	5–10 April 2001	Pak-EPA (2005)
Lahore (roadside monitoring)	TSP PM_10_	996 368	2003–2004	Ghauri et al. (2007)
Lahore (Pakistan Upper Atmospheric Research Commission Office)	PM_2.5_	209	December 2005 to February 2006	Biswas et al. (2008)
Lahore (University of Engineering and Technology, Lahore, UET)	PM_10_, OC, EC	459	February to March 2006	Zhang et al. (2008)
Lahore (Campus Bridge, Punjab University and Thokar Niaz Baig Chowk)	PM_10_ PM_2.5_ PM_1_ PM_10–2.5_	Average 286 222 210 340	November 2007	Ali et al. (2015)
Lahore (UET)	PM_10_ PM_2.5_	Elemental analysis	2007–2008	Schneidemesser et al. (2010)
Lahore (Township)	PM_2.5_ Metrological Parameter	Annual average 72.7 ± 55.2	2007–2008	Rasheed et al. (2015)
Lahore (UET)	PM_10_ PM_2.5_	Elemental analysis	2007–2008	Stone et al. (2010)
Lahore (19 different residential and commercial sites)	PM_10_	115	June to August 2012	Ashraf et al. (2013)
Lahore (UET Kala Shah Kaku site, UET Campus site and Lahore University of Management and Sciences)	PM_10_ Aerosol optical depth (AOD)	300 AOD 0.56–0.67	2014–2015	Khokhar et al. (2016)

The distribution and transport of PM in the atmospheric environments are markedly associated with meteorological parameters such as the wind speed, wind direction, relative humidity (RH), rainfall and ambient temperature (Pakbin et al. 2010). Therefore, PM concentrations and meteorological data should be evaluated statistically in order to develop correlations that can assist in identifying sources and thereby in the design of cost-effective emission control strategies (Ragosta et al. 2008). The data of ambient air quality are crucial in air resource management but are largely unavailable for rapidly growing cities of Pakistan. The analysis of a 5-year long-term data set provides significant insight into the factors that drive seasonal variations in PM, their relationship with meteorological parameters and criteria pollutants. This work could be used as an incentive to initiate other studies on trend analysis. It is also anticipated that the findings of this study would be of high relevance for designing and instituting future abatement strategies and emission regulations for the pollution control in rapidly developing cities such as Lahore.

The objective of this paper is to assess the long-term trend of fine particles PM_2.5_ at two different urban sites of Lahore (Pakistan) between 2007 and 2011. The trend of PM_2.5_ is compared with Pakistan National NAAQS and WHO guidelines. The seasonal changes in PM_2.5_ and their underlining reasons during weekdays and weekends, together with the correlation of PM_2.5_ with other pollutants and meteorological parameters, were also assessed. The AERONET data, backward trajectory and MODIS imageries were used to analyse the long-range transportation of PM and its seasonal contribution. The overall aim of these analyses is to form a basis for the development of appropriate regulatory strategies for limiting the exposure to ambient PM.

## Methodology

### Site description

**Fig. 1:**
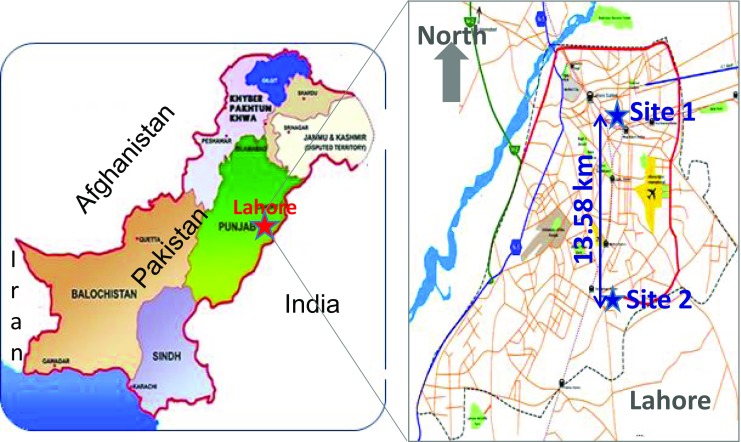
Location of ambient air quality monitoring sites: Townhall (*Site 1*) and Township (*Site 2*)

Lahore (31.320° N; 74.220° E) is the second most populated metropolitan area in Pakistan. The population of Lahore is approximately 9.44 million. There are ∼3.9 million motor vehicles and 2150 registered industries in the city (Bureau of Statistics 2015). The major industries in Lahore include the manufacturing of motor cars, motorcycles, steel, chemicals, pharmaceuticals, engineering products and construction materials. The aerosols over the sampling sites derive mainly from soil, road dust and industrial and vehicular emissions. Other anthropogenic sources include emissions from main highways, coal combustion and biomass burning (Biswas et al. 2008). Fixed-site ambient air quality monitoring stations are installed at two different urban locations of Lahore, namely Townhall and Township. Townhall represents a commercial area while the Township is representative of residential cum industrial areas, as shown in Fig. 1.

### Instrumentation

The hourly air quality monitoring data for 5 years between 2007 and 2011 were collected from the Environmental Protection Agency, Punjab (Lahore). Both ambient air quality monitoring stations were equipped with a number of instruments (i.e., combined wind vane, anemometer, thermohydrometer, solar radiation meter) to measure the metrological parameters and air pollutants, as summarized in Table 2. The routine checks of the instrument were carried out for their smooth operation on a weekly, monthly and annual basis to control the quality of the data. There were some gaps in the data due to power failure and routine maintenance (Table 2).

**Table 2:** Summary of instrument used for the measurements

Name of the instrument	Pollutant	Model	Method	Detection limit	Fraction of data available
CO monitor	CO	Horiba Ltd. Model APNA-370	Nondispersive infrared ray method (ISO4224)	0.1 ppm	55
NO_*x*_ monitor	NO_*x*_, NO, NO_2_	Horiba Ltd. Model APNA-370	Chemiluminescence (ISO7996)	0.5 ppb	50
SO_2_ monitor	SO_2_	Horiba Ltd. Model APSA-370	UV fluorescence method (ISO10498)	1 ppb	51
Ozone monitor	O_3_	Horiba Ltd. Model APOA-370	UV photometry method	0.5 ppb	50
Dust analyser	PM_2.5_	Horiba Ltd. Model APDA-370	β-Ray absorption method (ISO6349)	0–5 ppm	40

### Observation data and analysis

A data management and reporting software (IDA-ZRW) by HORIBA was used to collect and manage the data at both the ambient air quality monitoring stations. The statistical techniques such as Stata 3, R (Studio) and remote sensing tools such as AERONET were used further for the development of correlation of PM_2.5_ with meteorological and pollutant parameters. PM_2.5_ during weekdays and weekends and across 5 years was calculated, along with the exceedance factor, box plots, wind rose and bivariate polar plots. The satellite imageries from MODIS, backward trajectory and almucantar inversion were used to extract further data on the PM_2.5_ among different seasons, their sources and dispersion conditions. The almucantar inversion finds the minimum size intervals of PM from 0.439 to 0.992 μm (Dubuisson et al. 1996). This minimum size interval is used as a separation point among fine and coarse particles. It also estimates the effective radius, volume median radius, standard deviation and volume concentrations for both fine and coarse particles.

We estimated the annual exceedance factor (EF), and the percent decreases in PM_2.5_ were estimated to understand the exceedances over the regulatory limits. The annual EF was calculated by using Eq. (1):

1 $$ \mathrm{Annual}\ \mathrm{EF}=\frac{\mathrm{Observed}\ \mathrm{annual}\ \mathrm{mean}\ \mathrm{P}{\mathrm{M}}_{2.5}\ \mathrm{concentration}}{\mathrm{Standard}\ \mathrm{annual}\ \mathrm{mean}\ \mathrm{P}{\mathrm{M}}_{2.5}\ \mathrm{concentration}} $$

The air quality was categorized into four levels with respect to EF (i) critical pollution when EF >1.5, (ii) high pollution when EF is between 1.0 and 1.5, (iii) moderate pollution when EF is between 0.5 and 1.0 and (iv) low pollution when EF <0.5 (Kumar et al. 2014). The percent increase in daily and annual mean PM_2.5_ with respect to WHO guidelines is estimated using Eqs. (2) and (3):

2 $$ \mathrm{Daily}\ \mathrm{in}\mathrm{crease}\ \mathrm{in}\ \mathrm{P}{\mathrm{M}}_{2.5}\ \mathrm{concentrations}\ \left(\%\right)=\frac{\left(\mathrm{Observed}\ \mathrm{daily}\ \mathrm{mean}\ \mathrm{P}{\mathrm{M}}_{2.5}-\mathrm{Standard}\ \mathrm{daily}\ \mathrm{mean}\ \mathrm{P}{\mathrm{M}}_{2.5}\right)}{\mathrm{Standard}\ \mathrm{daily}\ \mathrm{mean}\ \mathrm{P}{\mathrm{M}}_{2.5}} \times 100 $$

3 $$ \mathrm{Annual}\ \mathrm{in}\mathrm{crease}\ \mathrm{in}\ \mathrm{P}{\mathrm{M}}_{2.5}\ \mathrm{concentrations}\ \left(\%\right)=\frac{\left(\mathrm{Observed}\ \mathrm{annual}\ \mathrm{mean}\ \mathrm{P}{\mathrm{M}}_{2.5}-\mathrm{Standard}\ \mathrm{annual}\ \mathrm{mean}\ \mathrm{P}{\mathrm{M}}_{2.5}\right)}{\mathrm{Standard}\ \mathrm{annual}\ \mathrm{mean}\ \mathrm{P}{\mathrm{M}}_{2.5}} \times 100 $$

## Results and discussion

### Temporal trend of PM_2.5_

**Fig. 2:**
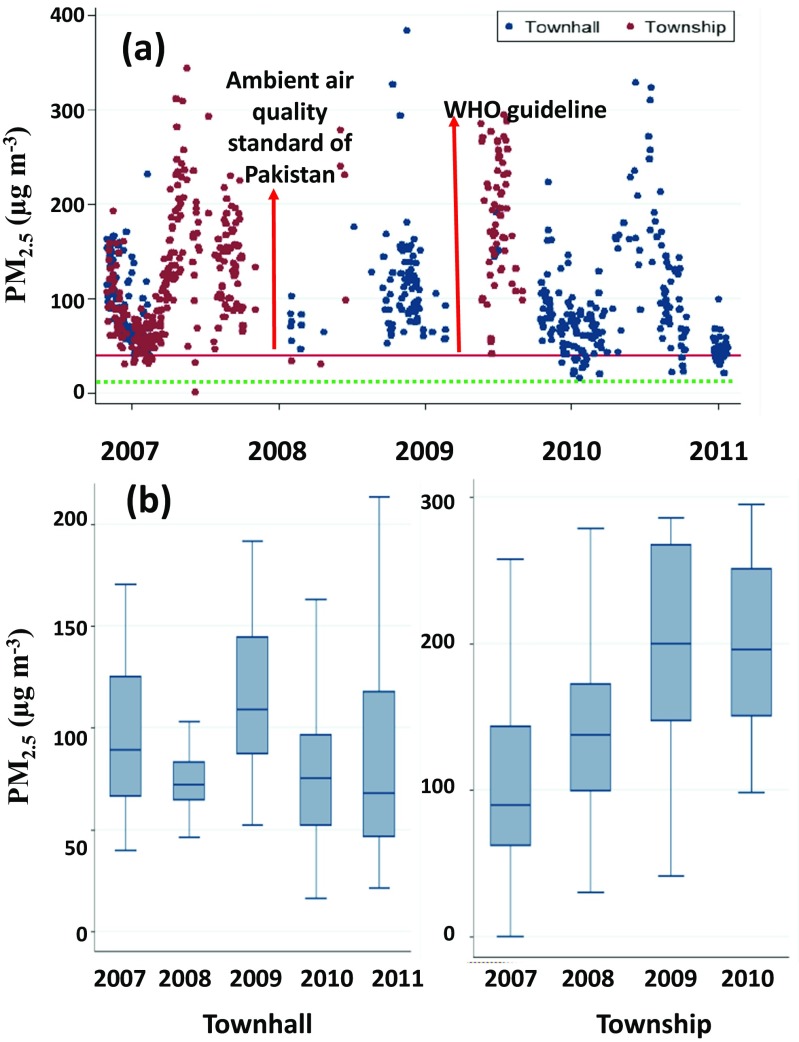
The trend of **a** daily and **b** annual means of PM_2.5_ at the studied sites in Lahore

Figure 2a shows the temporal trend of PM_2.5_ at both the sites between 2007 and 2011. The highest daily average concentration of PM_2.5_ was nearly the same at both sites, being 384 and 344 μg m^−3^ at the Townhall (16 May 2009) and Township (16 November 2007) sites, respectively (Fig. 2a). The annual average PM_2.5_ over the study duration at Townhall and Township was about 93 ± 23 and 180 ± 45 μg m^−3^, respectively. The annual average PM_2.5_ of both sides was 136 ± 34 μg m^−3^. Box plot presents the annual maximum, minimum and mean variation in PM_2.5_ during the study period (Fig. 2b). The annual mean of PM_2.5_ did not show an increasing trend over the years (Fig. 2b). One of the reasons is that the concentrations of PM_2.5_ were affected oddly by the local sources at Townhall site. For example, there was a construction activity of Metro transit system in Lahore during 2009 when the annual mean was noted to the highest. However, annual mean PM_2.5_ showed increasing concentrations with the time at the Township site, mainly because the sources contributing to PM_2.5_ were mainly stationary (industrial activities) that increased with the passage of time in this area.

**Fig. 3:**
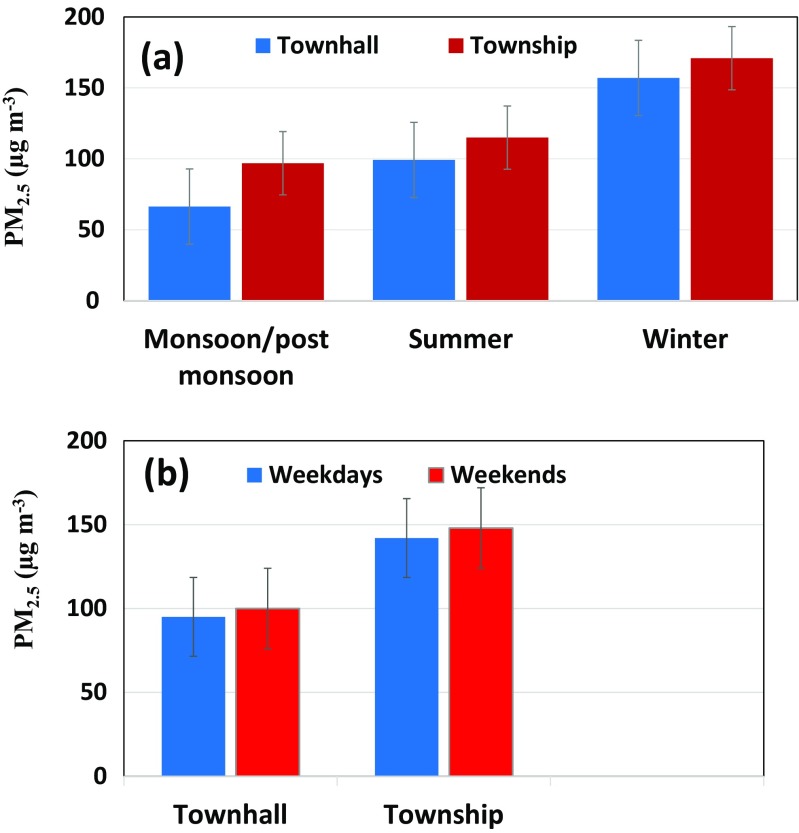
**a** Seasonal and **b** weekly trends of PM_2.5_ at the studied sites

The average minimum PM_2.5_ was 52 μg m^−3^ at Townhall in 2010 while the average maximum PM_2.5_ was 280 μg m^−3^ at Township in 2009. These concentrations were much higher than those observed in the European cities but near to PM_2.5_ found in Asian countries. For example, Ashraf et al. (2013) reported average annual PM_2.5_ in the capital (Islamabad) of Pakistan as 81.1 ± 48.4 and 93.0 ± 49.9 μg m^−3^ during 2007–2011, respectively. The similar case can be seen for the annual average concentration in the five most polluted megacities—Delhi (143.0 ± 17.8), Cairo (109.6 ± 27.7), Xi’an (102.2 ± 9.3), Tianjin (95.7 ± 7.7) and Chengdu (89.4 ± 14.4 μg m^−3^). Four of these most polluted cities in Asia in terms of PM_2.5_ were in Asia whereas only Cairo was in Africa. The five least polluted megacities in terms of PM_2.5_ were Miami (6.7), Toronto (8.4 ± 0.3), New York (9.1 ± 1.0), Madrid (9.9 ± 1.3) and Philadelphia (10.3 ± 1.0 μg m^−3^); among them, four were in USA and Canada and one (Madrid) in Europe (Cheng et al. 2016). The average annual PM_2.5_ of both sides of Lahore was 136.5 ± 34.1 μg m^−3^, which is clearly many fold higher than the USA and European cities and only comparable to Delhi with 143.0 ± 17.8 μg m^−3^. Table 1 presents the summary of the past relevant PM studies carried out in Pakistan. In general, PM_2.5_ and PM_10_ are many times higher than the WHO guidelines and NAAQS permissible limits. Schneidemesser et al. (2010) reported high levels of annual mean PM_10_ 340 μg m^−3^ for Lahore during 2007. Likewise, Stone et al. (2010) showed a maximum PM_10_ concentration of 650 μg m^−3^ on a typical polluted day during 2007. As for different seasons, the average PM_2.5_ during winter was ∼157 and 171  μg m^−3^ at Townhall and Township sites, respectively, followed by the corresponding values of ∼99 and 115 μg m^−3^ during summer and ∼66 and 97 μg m^−3^ during monsoon/post-monsoon (Fig. 3a). Winter, summer and monsoon/post-monsoon months were taken as November–February, March–June and July–October, respectively. The lowest PM_2.5_ was observed during monsoon/post-monsoon due to heavy precipitation as opposed to the highest PM_2.5_ during winter due to low inversion and stable atmospheric stability condition (Tiwari et al. 2013). The average concentration during the winter was about 53% higher than those during summer and almost double than those during the monsoon/post-monsoon. Similar seasonal trends were reported by Tiwari et al. (2013) in Delhi with daily mean PM_2.5_ in winter as 150.8 μg m^−3^, 70.9 μg m^−3^ during summer and 45.1 μg m^−3^ during monsoon.

The daily mean concentration of PM_2.5_ during weekends (Saturday–Sunday) was relatively higher than the weekdays (Monday–Friday) at both monitoring sites of Lahore. This is an interesting finding, which is opposite to many cities worldwide where much lower concentrations are usually reported during the weekends (Al-Dabbous and Kumar 2014; Yadav et al. 2014). For examples, the mean PM_2.5_ during the weekdays at the Townhall sites was measured as 95 μg m^−3^ as opposed to 100 μg m^−3^ during the weekends; the corresponding values were 142 and 148 μg m^−3^ at the Township site, respectively (Fig. 3b). The predominant reason for this interesting trend is that a relatively higher number of people living in surrounding suburban/rural areas visit Lahore for recreational purposes during the weekends, which is a typical feature of many Asian cities that result in increased traffic volume and in turn the PM_2.5_.

### Annual exceedances

**Fig. 4:**
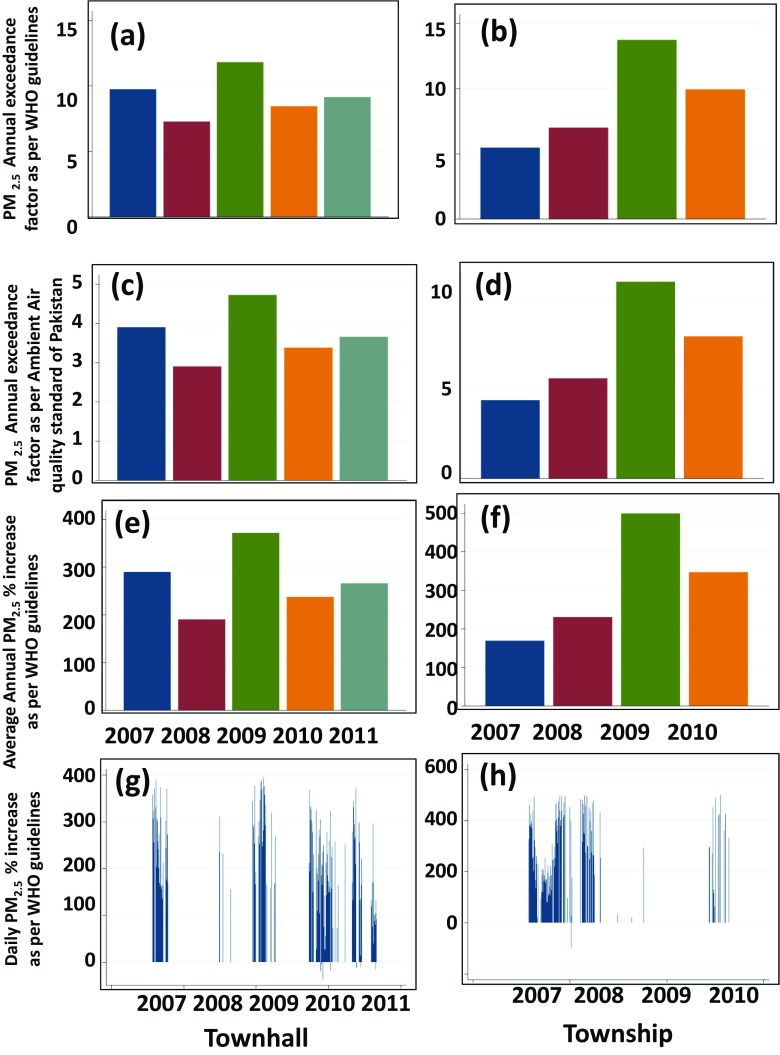
Annual exceedance factor as per WHO guideline (**a**, **b**) as per NAAQS of Pakistan (**c**, **d**) annual percentage increase (**e**, **f**) and daily percentage increase (**g**, **h**) as per WHO guidelines of PM_2.5_ at the studied sites in Lahore

The status of noncompliance at both sides of Lahore was measured by using annual EF, as described in “Observation data and analysis” section. The EFs for Townhall and Township with respect to WHO guidelines and NAAQS (Pakistan) lie within the range of 6–14 and 3–12, respectively (Fig. 4d). The result indicates the alarmingly high levels of PM_2.5_ on both sites of Lahore and categorizes them above critical pollution level (Kumar et al. 2014). The values for daily and annual percentage increases lie within the range of 100–500 and 180–500%, respectively (Fig. 4e–h). This shows that the noncompliance of PM_2.5_ with respect to WHO guidelines was mostly about 100–500% above on daily and annual basis, respectively. The sub-zero values in Fig. 4g, h represent the days when PM_2.5_ was less than the WHO guidelines.

Primary emissions of PM_10_ and PM_2.5_ decreased by 14 and 16%, respectively, in the EU-27 in 2011 compared with 2002–2011 levels (Ikeda and Tanimoto 2015). The reductions in the same period for the 32 member countries of the European Union were 9% for PM_10_ and 16% for PM_2.5_, respectively (Ikeda and Tanimoto 2015). In a WHO study, a total of 795 towns/cities from 67 countries were selected; 641 cities represent the high-income countries and 55 represent the middle- and low-income countries with available data of PM_10_/PM_2.5_ from 2008 to 2013. It was found that globally PM levels were increased by about 8%. The 90% of the low- and middle-income cities assessed exceeded annual WHO guidelines for PM_10_ and PM_2.5_. The worldwide future trends in PM_10_ and PM_2.5_ concentrations show a decrease in 30% of the regions as opposed to modest or increasing trend in the remaining 70% of the regions (WHO 2016). This study clear falls within the rest of 70% regions with increasing PM_2.5_ concentrations as is also the case with the most cities in developing countries (WHO 2016). The annual exceedances at the selected sites of Lahore were between 100 and 500% (Fig. 4e–h), indicating much higher concentrations compared with those reported in studies of European or high-income countries elsewhere (Ikeda and Tanimoto 2015; WHO 2016).

### Bivariate polar plots

**Fig. 5:**
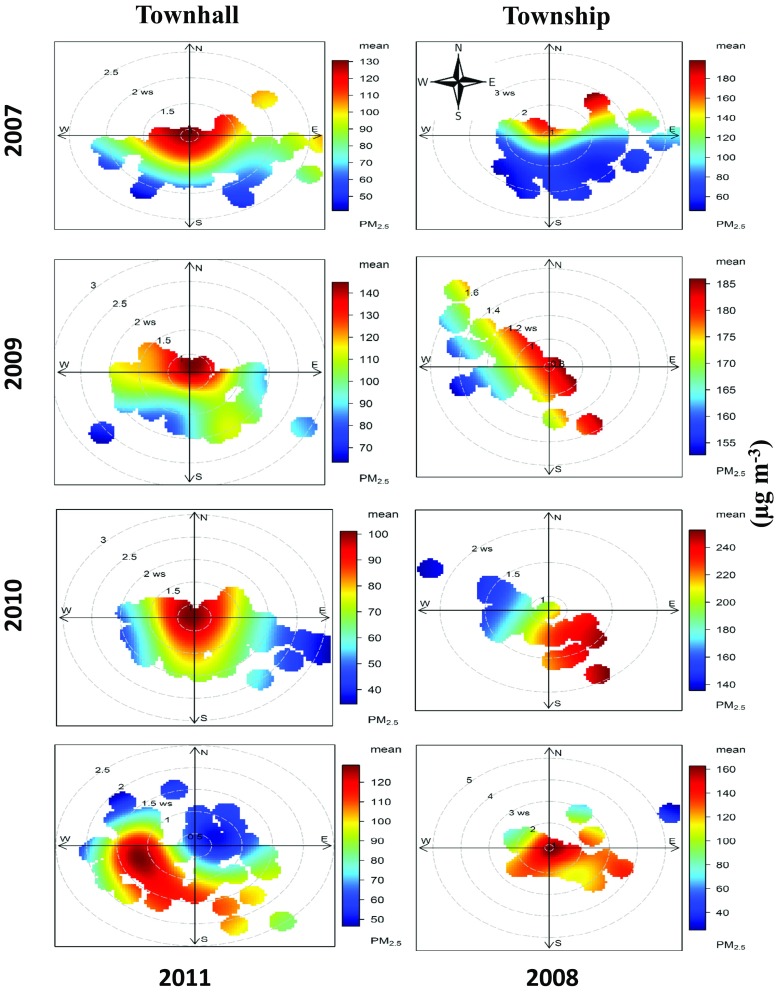
Annual bivariate polar plots for PM_2.5_ at both sites in Lahore

**Fig. 6:**
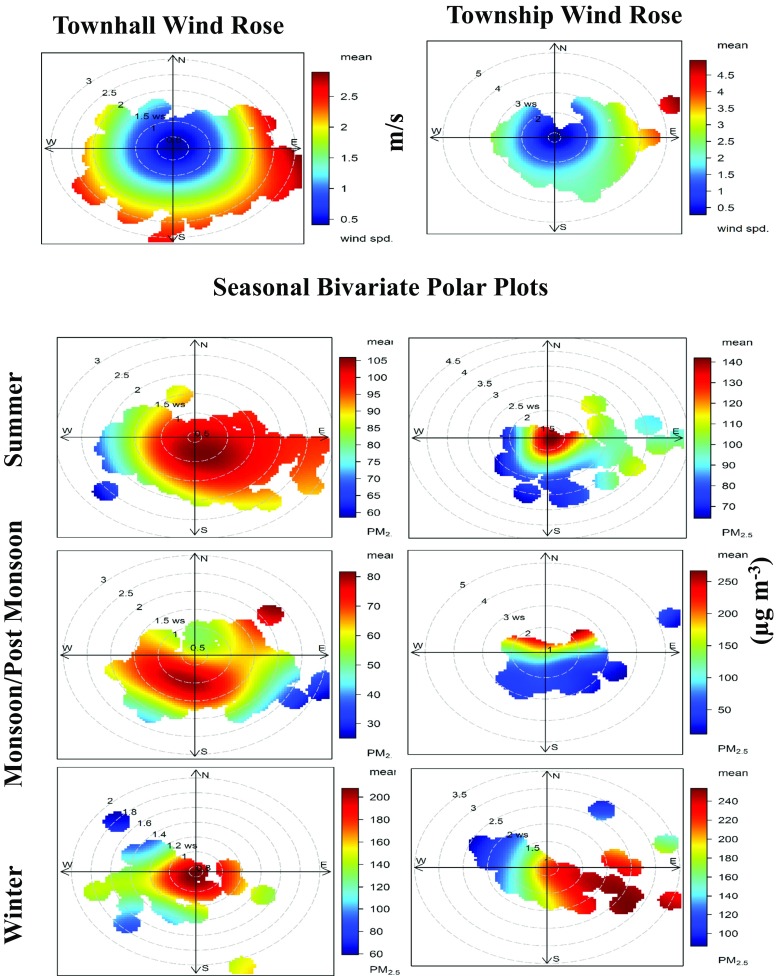
Bivariate polar rose plots for PM_2.5_ during different seasons at both sites in Lahore

Figures 5 and 6 show the bivariate polar plots for the annual and seasonal annual average PM_2.5_ concentrations for both the sites, respectively. A variation in concentrations, depending on the local wind direction and wind speed at the sampling locations, is clearly evident (Figs. 5 and 6). The similar methods of representing the air quality data have been adopted by past studies while assessing the long-term PM_2.5_ data (Azarmi et al. 2016; Mouzourides et al. 2015).

The colour scale of bivariate polar plots of PM_2.5_ shows the concentration, and the radial scale shows the wind speed. The concentration increases from the centre of the plot radially outwards in some cases while an opposite trend is seen in other cases. Bivariate polar plots of Townhall indicate that PM_2.5_ sources were mostly localized as depicted by high concentrations in the centre at low wind speeds, mainly contributed by the emissions from road vehicles (Fig. 5). A slight shift towards the southwest direction in monsoon/post-monsoon season at the Townhall was due to increased precipitation (Fig. 6). The annual bivariate polar plot of Townhall in 2011 showed a shift towards southwest due to intense construction activity of a 27-km-long bus rapid transit system in Lahore (Fig. 5); both the annual and seasonal bivariate polar plots for the Township indicate transport of PM_2.5_ to the site from the presence of industrial areas in the east and southeast direction of air monitoring station (Figs. 5 and 6).

### Correlation of PM_2.5_ with the criteria pollutant and meteorological parameters

**Fig. 7:**
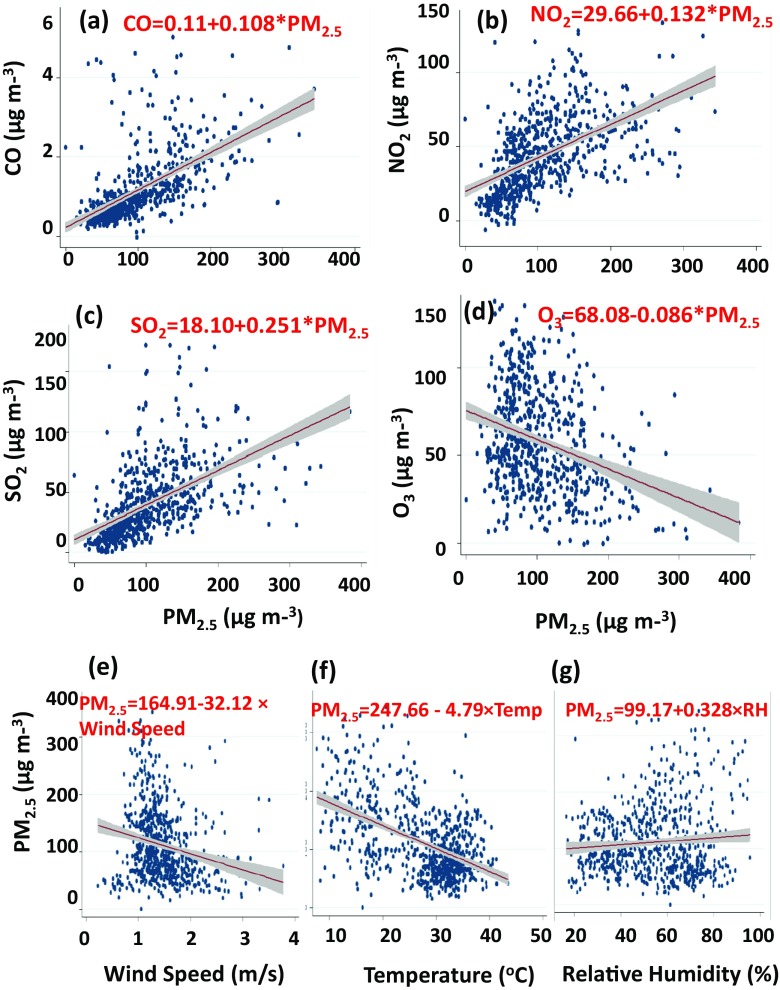
Correlation of PM_2.5_ with CO, NO_2_, SO_2_ and O_3_ (**a**–**d**) and with wind speed, temperature and relative humidity (**e**–**h**) during the studied period at Townhall

Regression analysis was used to assess the correlation between PM_2.5_ and NO_2_, CO, O_3_ and SO_2_ (Fig. 7a–d). The positive correlation was found among NO_2,_ CO, SO_2_ and PM_2.5_ with 95% confidence interval. Diesel combustions from heavy duty vehicles, electricity generators and industrial emissions were considered to be a major source of both CO, SO_2_ and NO_2._ The association between CO, SO_2_, NO_2_ and PM_2.5_ was significantly positive, suggesting that they were contributing to the production of PM_2.5_. On the other hand, a negative correlation of PM_2.5_ with O_3_ suggests that O_3_ was increased when PM_2.5_ was decreased. Previous studies (Ashraf et al. 2013; Rasheed et al. 2015) reported the similar correlations among PM_2.5_ and NO_*x*_, CO, O_3_ and SO_2_ in different cities of Pakistan, indicating the consistency of our results with the past observations.

The correlations among the significant meteorological parameters such as wind speed, ambient temperature, RH and PM_2.5_ show a negative correlation with temperature (Fig. 7e) and wind speed (Fig. 7f) and no correlations with the RH (Fig. 7g). This demonstrates the fact why PM_2.5_ concentrations were much higher in winter than in summer (Fig. 3a) due to a decrease in temperature and wind speed. Such higher levels raise a number of concerns including reduced visibility affecting the speed of on-road vehicles and the increased cases of both chronic and acute respiratory and cardiovascular health problems in the region, as discussed by previous studies (Tiwari et al. 2013; Yin et al. 2016).

### MODIS fires hotspots and the effect of transboundary pollution

**Fig. 8:**
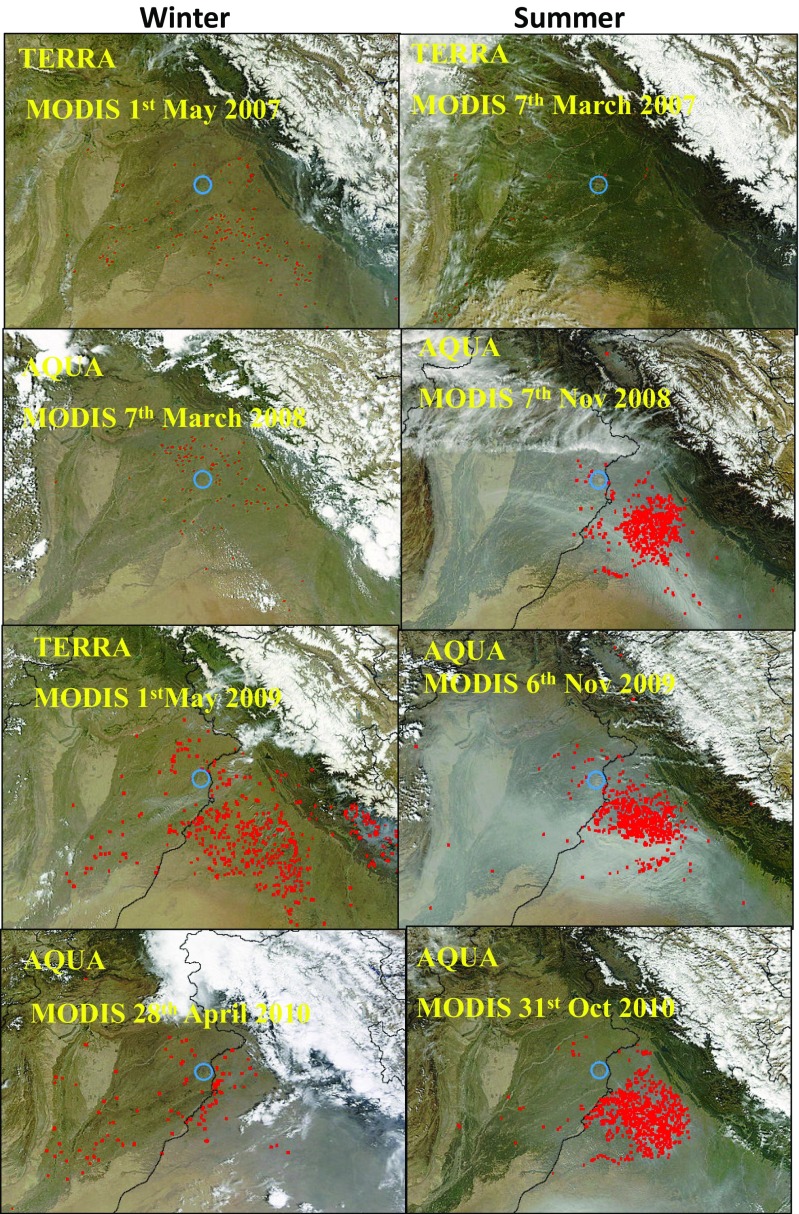
MODIS Terra/Aqua imageries in summer and winter seasons of Lahore

The MODIS Aqua/Terra imagery data were used for the identification of pollution hotspot in the study area during the summer and winter seasons (Fig. 8). The red spots indicate the major sources of air pollution. The predominant winds of Lahore come from west and northwest in the winter season whereas from the southeast during the summer and post-monsoon seasons (Fig. 6). MODIS Terra/Aqua imageries in summer and winter seasons of Lahore were used to assess the trans-boundary movement of air pollution. The transport of air pollution during November to February was not so significant because the average mean wind speed during these months was ∼1.5 m/s compared with ∼3.5 m/s between March and October. A recent study by Rasheed et al. (2015) included the back-trajectory analysis of four major cities of Pakistan and reported that the air masses originating from western India were from the states of Gujrat, Rajasthan and Punjab with sources generating PM_2.5_ such as coal-fired power plants, industries and vehicular emissions, which contribute to air pollution of Lahore (Singh and Kaskaoutis 2014; Rasheed et al. 2015). In addition, wheat harvesting during March–April and dry winter climatic conditions also play an important role in elevated PM_2.5_ values during the months of October–November in Lahore.

### Size distribution of aerosol particles

**Fig. 9:**
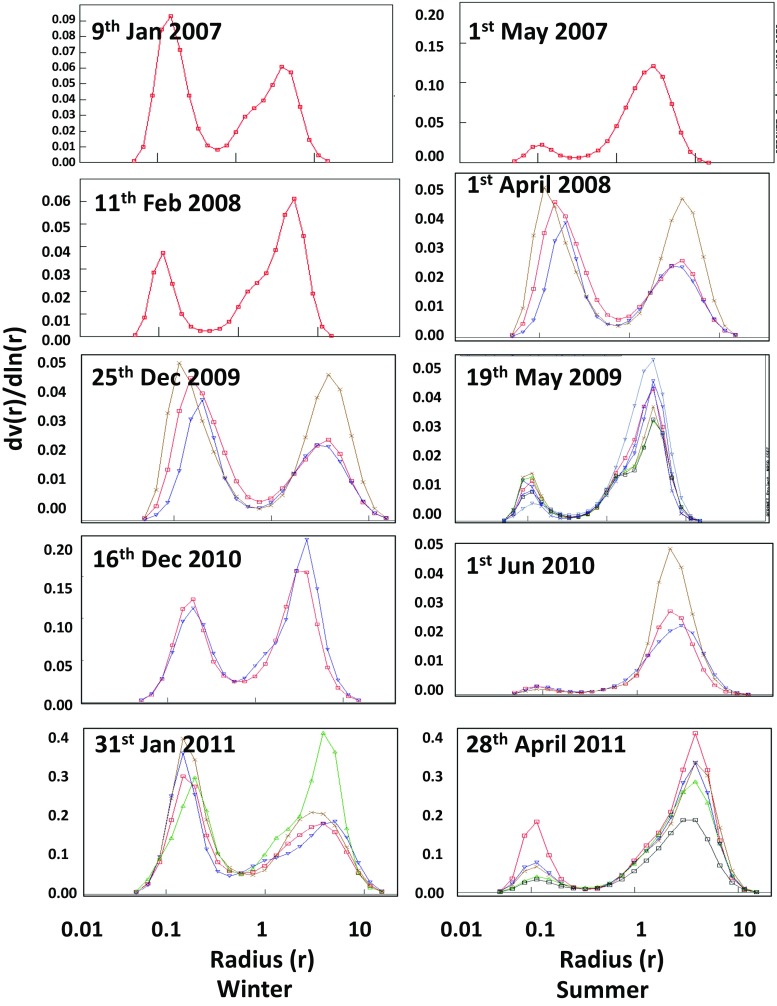
Relative particle size distribution in winter and summer seasons of Lahore

The almucantar inversion aerosol optical property retrieved from AERONET data was used to find out the relative particulate size difference of fine and coarse particles during winter and summer seasons of Lahore during the study period (Fig. 9). The relative difference in PM_10_ was much higher in summer than winter. The similar results were reported by Ali et al. (2013) on the size distribution of coarse particles in Lahore. They found PM_10_ to be three times higher in summer than in winter and fall seasons. However, fine mode particles did not show any substantial difference in concentration during all the four seasons. A similar trend was observed by Dey et al. (2004) while analysing the effect of dust storms on seasonal optical properties of the Indo-Gangetic region. The increased wind speed caused gale and wind storms during summer, besides an increase in the relative difference of PM_10_ among winter and summer seasons. The AERONET almucantar inversion data present the substantial relative difference in PM_10_ whereas the marginal substantial difference in PM_2.5_ of winter and summer seasons, opposed to a relative difference of ground-based data of PM_2.5_ as shown in Fig. 3a.

## Summary and conclusions

We assessed the temporal trend of fine PM (PM_2.5_) over a period of 5 years in Lahore. The annual mean PM_2.5_ concentrations were found to be increasing at Township site and show no clear trend at the Townhall site during the study period. Our findings show that the levels of PM_2.5_ reach to their highest levels during the winter season. For example, the highest daily mean PM_2.5_ measure at Townhall and Township was found to be 389 and 354 μg m^−3^, respectively.

The annual average minimum PM_2.5_ was found to be 52 μg m^−3^ at Townhall during 2010 while the average maximum PM_2.5_ was 280 μg m^−3^ at Township during 2009. PM_2.5_ crossed 98% daily and 100% annual permissible limits of NAAQS and WHO guidelines at both sites of Lahore. The average concentrations during the winter were found to be about 53% higher than those during summer and almost double than the monsoon/post monsoon, mainly due to a decrease in temperature and stagnant climatic conditions. Seasonal air quality trend of Lahore from 2007 to 2011 was analysed and found that the highest annual mean PM_2.5_ in winter was 157–171 μg m^−3^, summer 99–115 μg m^−3^ and monsoon/post-monsoon 66–97 μg m^−3^ at Townhall and Township, respectively.

PM_2.5_ during weekdays was usually less by up to 4% than weekends. The annual EF of PM_2.5_ with respect to WHO guidelines lies within the range of 3–14 and 6–12 with respect to NAAQS of Pakistan at Townhall and Township sites, respectively. The daily and annual % increases lie in the range of 100–500% with respect to WHO guidelines at both monitoring sites of Lahore.

The sources contributing to PM_2.5_ at the Townhall site were mostly localized as opposed to Township where there is the influence of transported emissions from the adjacent industrial sites. Correlation of PM_2.5_ with CO, NO_2_ and SO_2_ was positive and negative with O_3_. However, the correlation of PM_2.5_ with meteorological parameters such as temperature and wind speed was negative and nonsignificant with RH. Retrieved MODIS Aqua/Terra imageries, together with predominant wind direction, showed the influence of transboundary air pollution from India towards Lahore during the months of March to October as opposed to an opposite trend during the months of November to February when the long-range transport of PM_2.5_ is from Lahore to India.

This study contributes to understanding the long-term trend of PM_2.5_ in the urban environment of Lahore. Our findings are important to understanding the surrounding sources and underline the factors that bring the seasonal variability in PM_2.5_. Further studies require the monitoring at a greater number of sites to broaden the understanding of spatial variability across the city along with a physicochemical analysis of the fine particles.
